# Monitoring self-reported adherence to antiretroviral therapy in public HIV care facilities in Brazil: A national cross-sectional study

**DOI:** 10.1097/MD.0000000000009015

**Published:** 2018-05-25

**Authors:** Maria Altenfelder Santos, Mark Drew Crosland Guimarães, Ernani Tiaraju Santa Helena, Cáritas Relva Basso, Felipe Campos Vale, Wania Maria do Espírito Santo Carvalho, Ana Maroso Alves, Gustavo Machado Rocha, Francisco de Assis Acurcio, Maria das Graças Braga Ceccato, Rogério Ruscitto do Prado, Paulo Rossi Menezes, Maria Ines Batistella Nemes

**Affiliations:** a Faculty of Medicine of University of Sao Paulo, Department of Preventive Medicine, São Paulo, São Paulo; b Federal University of Minas Gerais, Belo Horizonte, Minas Gerais; c University of Blumenau, Blumenau, Santa Catarina; d Federal University of São João Del-Rei, Divinópolis, Minas Gerais, Brazil.

**Keywords:** antiretroviral therapy, Brazil, health services research, HIV/AIDS, national survey, nonadherence

## Abstract

**Introduction:**

Patient adherence to antiretroviral therapy (ART) is critical for HIV treatment success. Monitoring rates of adherence in public HIV outpatient care facilities can improve outcomes in Brazil where ART is universally available.

**Methods:**

We conducted a national cross-sectional survey of ART adherence in 2010. Participants were selected using a multistage probability sample. First, HIV outpatient care facilities were stratified according to 7 Organizational Quality Classification (OQC) groups and regions. Second, 1 or 2 facilities were selected per region for each OQC group. Finally, patients were randomly selected at each facility. In a first component, patients were invited to answer to a web-based questionnaire (*WebAd-Q*), a validated self-reported tool that includes 3 questions on adherence to ART in the past 7 days (time scheduling—timing, drug regimen—medication, and pill counts—dose), herein named indicators of potential nonadherence (IPN). In addition, a subsample of participants were interviewed in order to obtain further data on sociodemographic and clinical characteristics (second component). The proportion of each IPN was estimated using weighted data to account for the sampling design with 95% confidence interval (CI) and descriptive analysis was carried out.

**Results:**

Fifty-five facilities were chosen and 2424 patients completed the *WebAd-Q* in the first component of the study, while 598 patients were interviewed for the second component. The weighted proportions of the IPN were 50.9%, 31.8%, and 19.5%, for timing, medication, and dose, respectively, while11.7% had all 3 indicators, varying from 5.9% in the Southeast and 21.9% in the Northeast regions. Overall, 61.1% of the patients had at least 1 IPN (95% CI: 58.5–63.7%). Patients reporting depression symptoms, illicit drug use and those who missed medical appointments had worse nonadherence outcomes.

**Conclusions:**

Overall, there was a high proportion of all indicators IPN and timing was the main component associated with low adherence. Although these indicators may not necessarily indicate individual nonadherence, they represent a worrisome scenario in the public Brazilian HIV care facilities. On a routine basis, these facilities can identify gaps in providing counseling and ART orientation to their clientele and develop innovative strategies to prevent nonadherence.

## 1 Introduction

Patient adherence to antiretroviral therapy (ART) is critical to ensure the effectiveness of HIV treatment. Since the advent of highly active antiretroviral therapy (HAART), high levels of adherence have been consistently associated with improved virologic, immunologic, and clinical outcomes,^[1–3]^ with consequent increase in survival^[4–6]^ and quality of life.^[7,8]^

Even though recent and more potent regimens indicate the possibility of achieving satisfactory outcomes at moderate levels of adherence,^[9,10]^ the assumption that higher adherence levels lead to improved outcomes remain valid.^[11–13]^ However, ART consists of a lifelong, complex treatment, which often involves side effects. As a result, patient adherence is inconsistent and tends to decline over time.^[6,14]^ Even with more tolerable and simpler regimens currently available, adherence remains challenging for HIV programs worldwide.^[15]^

In view of this context, recent international guidelines for the organization of HIV care highly recommend routine monitoring of adherence in clinical settings.^[16–19]^ Adherence monitoring is critical to guide treatment planning and prevent virologic failure during medical follow-up.^[20]^ In addition, routine adherence measurement is a valuable tool in managing HIV programs in public health facilities, including the implementation of interventions to promote and support adherence and routine evaluation of program and facilities’ performance.^[21–24]^ Furthermore, adherence assessment is a key element in the implementation of treatment-as-prevention strategies along with efforts to improve outcomes in all stages of the HIV treatment cascade.^[25]^

Currently, there are approximately 405,000 individuals receiving ART at approximately 1024 human immunodeficiency virus (HIV)/acquired immune deficiency syndrome (AIDS) referral facilities through the Brazilian universal public health system.^[26,27]^ This number should increase in the coming years considering the introduction of treatment as prevention strategies and the constant updates in treatment guidelines to include HIV-infected individuals regardless of CD4+ cell counts.^[28,29]^

Despite efforts of the Department of STI, HIV/AIDS and Viral Hepatitis, Ministry of Health (DIAHV/MoH) to disseminate strategies to promote and sustain patients’ adherence,^[30,31]^ its assessment is not a standard practice in most facilities and Brazil still lacks standardized, valid, and feasible measures for routine use in public HIV care facilities.^[32,33]^

To address this gap, a self-reported tool to monitor ART and screen for potential nonadherence in the Brazilian public HIV care facilities has been recently developed and validated—the *Qualiaids Web Adherence Questionnaire* (*WebAd-Q*).^[34]^ The *WebAd-Q* was developed to be a practical tool for routine use, capable of addressing different dimensions of adherence, that is, scheduling, drug regimen, and dose. In the validation study, the *WebAd-Q* measures were associated with viral load, and performed well in comparison to concurrent measures (i.e., electronic monitoring, pill counting, self-report interview).^[34]^

The *WebAd-Q* focus on indicators at the facility level, rather than on medically oriented individual adherence. It allows for periodic screening of a healthcare facility clientele so that staff managers can continuously assess service performance and define effective strategies to promote adherence in each facility. In this paper, we present overall descriptive results of a national application of the *WebAd-Q* in the Brazilian public HIV care facilities.

## 2 Methods

### 2.1 Study design

National cross-sectional survey of adherence conducted in 2010 among HIV-infected patients receiving ART in public HIV outpatient care facilities in Brazil. Patients should be 18 years old or over and facilities should have been registered with the DIAHV/MoH as of 2007 (N = 636).^[35]^ Pregnant women were excluded from the study. The study was divided into 2 components: Overall assessment of adherence; application of a semi-structured interview in a subset of participants from the first component in order to assess potential determinants of nonadherence.

### 2.2 Sampling procedures

A 2-stage probability sampling procedure was used in the survey: selection of facilities; and selection of participants. For the first stage, 2 characteristics were taken into account, an Organizational Quality Classification (OQC) of each facility^[35]^ and the 5 main Brazilian geographical regions (North, Northeast, Central-West, South, and Southeast), plus the States of São Paulo and Rio de Janeiro which were treated separately due to their large contributions to AIDS cases in the country.^[36]^ The OQC was determined by a previous national assessment of structural (e.g., access to care, infrastructure, human resources, availability of medications, exams, and referral service) and process characteristics (e.g., reception of new patients, counseling, medical and nurse care, multidisciplinary team, adherence-focused activities, work flows and protocols, coordination, professionals’ training and updating, data monitoring, evaluation and planning, patient and civil society participation), ranging from Group 1 (highest OQC) to Group 6 (lowest OQC).^[35]^ A seventh group includes facilities with unknown OQC.^[35]^

### 2.3 Selection of facilities

Within each OQC group we selected 1 or 2 facilities from each region according to the distribution of patients receiving HIV care. One facility was selected when the region contained up to 20% of patients receiving HIV care within each OQC group. Otherwise, 2 facilities were selected. The facilities were selected using simple random sampling proportional to the size of each facility within each region and OQC group. Overall, 55 facilities were selected, ranging from 5 to 9 per OQC group.

### 2.4 Selection of participants

We anticipated a sample size of 336 participants per OQC group plus an estimated nonparticipation rate of 12.0% considering an estimated prevalence of adherence of 70.0% (95% CI: 65.0–75.0%) and a significant level of 0.05. Once the facilities were selected, we used simple probability sampling proportional to the number of patients receiving ART within each OQC group. The minimum sample size for each facility was set at 5 participants, yielding a total of 2646 patients to be recruited for the first component of the study. A subset of 598 participants was randomly selected proportional to the number of patients under ART in each region for further interview during the second component.

### 2.5 Data collection and measures

Treatment adherence was assessed using the *WebAd-Q*, a previously validated self-reported tool developed to monitor an overall measure of adherence in Brazilian public HIV care facilities.^[34]^ Two healthcare providers from each selected facility participated in a 1-day training regarding the patients’ recruitment process and invitation to participate in the study according to standardized procedures. Patients selected for the first component answered to the *WebAd-Q* in private rooms in the healthcare facilities, and no personal data were collected.

The *WebAd-Q* is an anonymous self-administered web-based questionnaire designed as a computer animated cartoon. It contains 3 questions, each one corresponding to one dimension of ART adherence: Timing: “In the last 7 days, have you taken any of your regimen drugs at times other than those scheduled by your doctor?”; Medication: “In the last 7 days, have you failed to take any of your regimen drugs?”; and Dose: “In the last 7 days, have you taken less or more pills of any of your regimen drugs?” Possible answers were “Yes,” “No,” or “I do not know/Do not recall.” The answer “No” is an indicator of adherence and the answers “Yes” and “I do not know/Do not recall” are indicators of potential nonadherence (IPN).

For the second component (subset sample) a brief questionnaire was applied by face-to-face interviews. The interviews were conducted by trained members of the research team. Data on sociodemographic (age, gender, skin color, socioeconomic class), on ART (e.g., side effects, difficulties with treatment, missed medical appointments, time since HIV diagnosis and ART), substance use (e.g., illicit drugs and alcohol). Self-rated quality of life and anxiety and depression symptoms were also obtained. Depression and anxiety were assessed by the Hospital Anxiety and Depression scale (HAD) previously tested and validated in Brazil^[37]^ and quality of life by the WHOQOL-HIV.^[38]^

### 2.6 Statistical analysis

In this report we present descriptive data on the first component and selected characteristics of the second component of the study. We analyzed the frequency distribution of the answers to the *WebAd-Q* according to each dimension with 95% confidence intervals (CIs). We also analyzed the accumulation of the 3 dimensions (i.e., timing, medication, dose), as follows: nonadherence in only 1 dimension; nonadherence in any combination of 2 dimensions; nonadherence in all 3 dimensions; and nonadherence in at least 1 of the 3 dimensions. Categories 1 and 2 were irrespective of which dimension reported.

The proportions were weighted to account for the sampling design (i.e., the probability of facilities and patients being selected) using SAS statistical software by means of the complex sampling analysis procedure (SURVEYFREQ). The OQC groups were considered the strata level.

### 2.7 Ethics statement

This study was approved by the Research Ethics Committee from the Faculty of Medicine, University of São Paulo (Protocol no.: 1140/09) and by local Ethical Review Boards when requested by the healthcare facilities. Participants who agreed to participate in the study provided written informed consent. Confidentiality of participants’ identity was maintained. For the second component an additional written informed consent was also obtained.

## 3 Results

Fifty-five public HIV care facilities from 7 Brazilian regions participated in the study. A total of 2604 patients were invited to answer the *WebAd-Q* and 2424 (93.1%) completed the questionnaire during the first component. Of those invited to participate in the study, 97 (3.7%) declined and 54 (2.1%) did not meet the eligibility criteria. Lack of time was the main reason for declining to participate. Problems with administration of the questionnaire (incomplete or duplicated data) accounted for 29 (1.1%) exclusions. Table 1 shows the distribution of the sample according to facilities and patients.

**Table 1:**
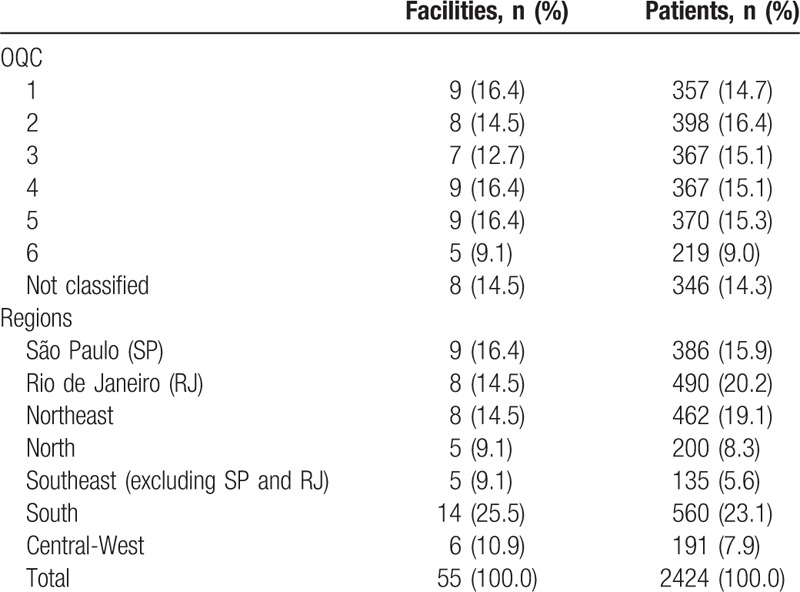
Sample distribution of facilities and patients according to the Organizational Quality Classification (OQC) and regions.

Overall, 50.7% of the patients reported timing nonadherence while lower proportions were observed for medication and dose (32.2% and 20.3%, respectively) (Table 2). Although some heterogeneity was found for regions (42.2–59.4% for timing; 26.4–34.3% for medication; 12.9–30.5% for dose) and OQC (42.3–60.3% for timing; 21.9–36.8% for medication; 3.4–23.3% for dose), there was no indication of statistically significant differences (CI overlapping).

**Table 2:**
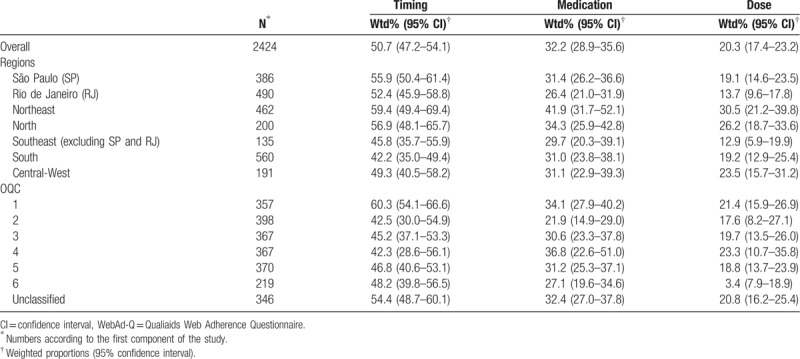
Weighted proportions of the *WebAd-Q* dimensions (nonadherence indicators) by regions and Organizational Quality Classification (OQC), Brazil 2010.

When taking the number of indicators reported, 61.1% (95% CI: 57.8–64.4%) of the patients reported at least 1 dimension (Table 3) and decreasing proportions were shown for only 1, 2, or all 3 dimensions, respectively. However, only Rio de Janeiro and the Northeast regions showed proportions with nonoverlapping CI for the presence of all 3 dimensions.

**Table 3:**
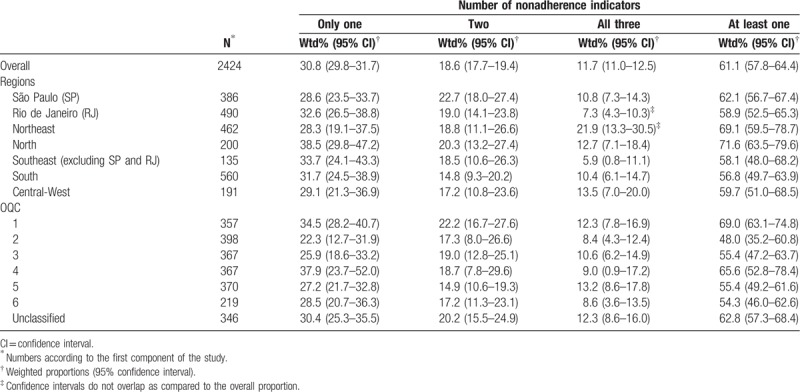
Weighted proportions according to the number of nonadherence indicators by regions and Organizational Quality Classification (OQC), Brazil 2010.

The analysis of the subset sample (second component), indicated that most variables had similar trends for each dimension of nonadherence (Table 4). Higher proportions were found among women, those at younger age, nonwhite, with lower schooling and lower social class, with moderate to severe depression or anxiety, low self-rated quality of life, and those who reported current alcohol or illicit drug use. In addition, participants with more recent HIV diagnosis and ART, who reported missing any medical appointment in the last six months and adverse reactions also showed higher proportions of the nonadherence indicators. However, only missing medical appointments showed nonoverlapping CI in each of the 3 dimensions, while depression symptoms and illicit drug use showed nonoverlapping CI for the timing dimension only. Similar results were also seen for those with nonadherence in at least 1 of the dimensions, but only depression symptoms and missing appointments were statistically different (Table 5).

**Table 4:**
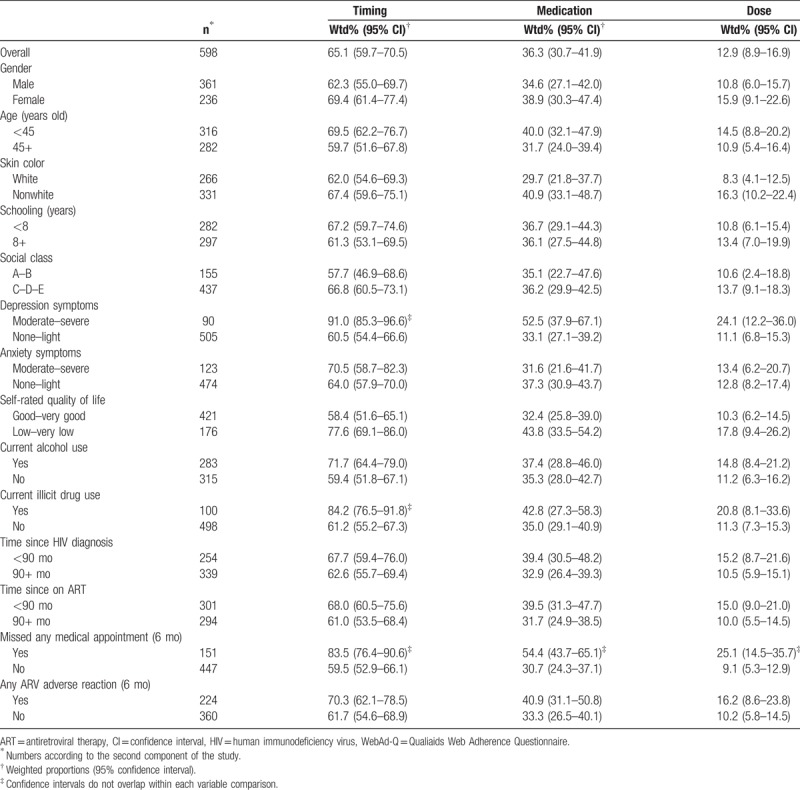
Weighted proportions of the *WebAd-Q* dimensions (nonadherence indicators) according to selected characteristics of the subset sample (n = 598), Brazil 2010.

**Table 5:**
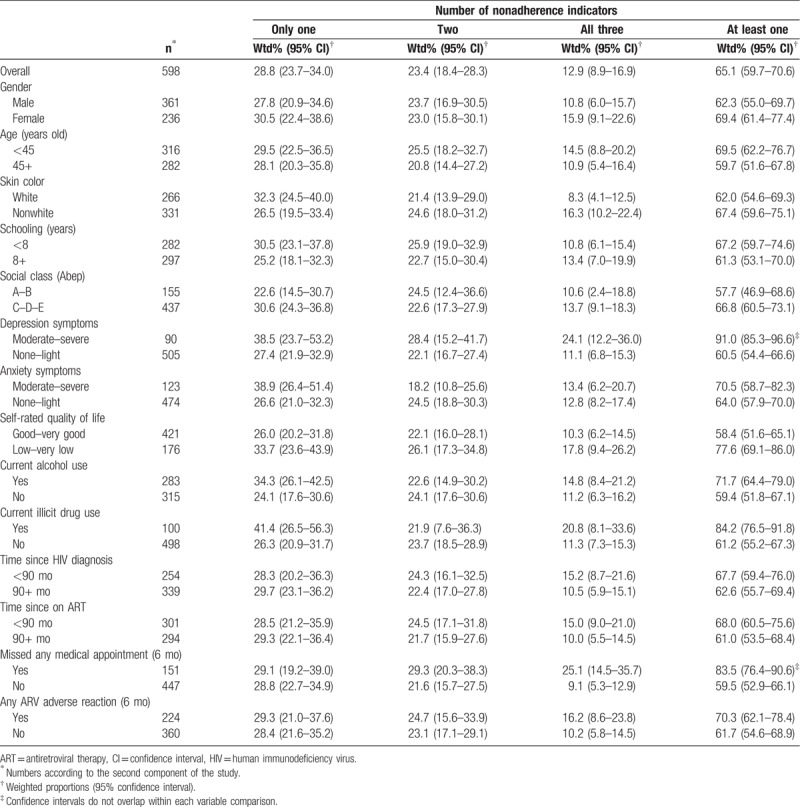
Weighted proportions according to the number of nonadherence indicators by selected characteristics of the subset sample (n = 598), Brazil 2010.

## 4 Discussion

The national application of the *WebAd-Q Questionnaire* showed high proportions of nonadherence indicators in Brazil, either analyzing the 3 dimensions separately or in accumulation—61.1% had at least 1 nonadherence indicator and timing was the most common one, suggesting that complying with recommended ART schedules is still a challenge in Brazil.

National data on nonadherence in Brazil are still scarce. Before the present study, only 1 study has estimated national measures of adherence in Brazil, in 2001.^[39]^ In that report, individual adherence was measured using a more traditional concept of nonadherence, that is, patients who reported taking less than 95% of the prescribed regimen during the 3 days before the interview. Approximately one-quarter of the patients were considered nonadherent (24.9%; 95% CI: 23.0–26.9%). Other studies of ART adherence among adult patients in Brazil show large variations in nonadherence rates across the country, varying from 10.7% to 86.0%,^[33,40–57]^ depending on study design (e.g., cross-sectional, longitudinal), target populations (e.g., general population, alcohol and drug users, patients initiating ART), measurement tools (e.g., self-reports, pharmacy records, pill counts, patient records), and adherence definitions (e.g., percentage of doses taken, time between medication refills). Similar to international studies,^[58,59]^ this methodological heterogeneity limits proper comparison among studies.

Results from the subset sample are also corroborated by findings from studies approaching individual barriers to ART adherence, including depression symptoms and illicit drug use.^[60]^ Also, missing medical appointments has been consistently associated with worse HIV treatment outcomes^[61–64]^ which is in itself an important indicator of nonadherence.^[22,23]^ Individual factors associated with nonadherence may vary across facilities and regions, thus demanding specific measures suitable for each scenario. However, contrary to our previous findings,^[65]^ there was no clear indication of the influence of OQC over the nonadherence indicators in this study and differences found between regions are still inconclusive. Further analyses are needed to better understand the influence of facility and patient characteristics on the nonadherence indicators.

The *WebAd-Q* methodology provides data for continuous evaluation of HIV care facilities by measuring ART nonadherence indicators in different dimensions during the 7 days previous to a regular follow-up medical visit. If periodically obtained, *WebAd-Q* measures could help guide providers in respect to which adherence dimensions tend to be more problematic among their clientele.

The findings of our study must be interpreted with caution due to potential limitations. This was a cross-sectional sample, and direct causal associations and changes in the nonadherence indicators over time among facilities and regions cannot be observed. However, these can be partially overcome if facilities decide to apply the *WebAd-Q* on a regular basis, or through periodic surveys. In addition, we should note that the *WebAd-Q* may not be a direct measure of individual nonadherence. However, it has been shown to be associated with viral load (i.e., indicators of nonadherence were associated with higher viral load) and with traditional adherence measures^[34]^ and, thus, can be considered a robust and valid tool for screening nonadherence in HIV care facilities in Brazil. Finally, although the patients answered the *WebAd-Q* anonymously, adherence measures collected in a standardized research protocol may not entirely correspond to measures obtained in routine care, and additional data should be obtained to corroborate the findings, including CD4+ cell counts, viral load, and ARV resistance assays.

In conclusion, the results obtained in this national study represent a worrisome scenario in the public Brazilian HIV care facilities with regard to adherence to ART. On a routine basis, these facilities can identify gaps in providing counseling and ART orientation to their clientele and develop innovative strategies to prevent nonadherence, including training and update workshops of staff.

## Author contributions

**Conceptualization:** M.A. Santos, M.I.B. Nemes,

**Funding acquisition:** M.I.B. Nemes.

**Methodology:** M.I.B. Nemes.

**Writing – original draft:** M.A. Santos.

**Writing – review & editing:** M.A. Santos, E.T. Santa Helena, F.C. Vale, W.M.d.E.S. Carvalho, A.M. Alves, G.M. Rocha, F.d.A. Acurcio, M.d.B. Ceccato, M.I.B. Nemes.

## Acknowledgments

The authors gratefully acknowledge review suggestions of Paul D. Cleary and the T32 Interdisciplinary HIV Prevention Training Program—Center for Interdisciplinary Research on AIDS (CIRA), Yale School of Public Health.
